# Superconducting-Gap Anisotropy of Iron Pnictides Investigated via Combinatorial Microwave Measurements

**DOI:** 10.1038/s41598-020-63304-0

**Published:** 2020-04-27

**Authors:** Tatsunori Okada, Yoshinori Imai, Kentaro Kitagawa, Kazuyuki Matsubayashi, Masamichi Nakajima, Akira Iyo, Yoshiya Uwatoko, Hiroshi Eisaki, Atsutaka Maeda

**Affiliations:** 1 Department of Basic Science, University of Tokyo, Meguro, Tokyo 153-8902 Japan; 2 Institute for Materials Research, Tohoku University, Sendai, Miyagi 980-8577 Japan; 3 Department of Physics, Tohoku University, Sendai, Miyagi 980-8578 Japan; 4 Institute for Solid State Physics, University of Tokyo, Kashiwa, Chiba 277-8581 Japan; 5 National Institute of Advanced Industrial Science and Technology, Tsukuba, Ibaraki 305-8568 Japan; 6 Present Address: Department of Physics, University of Tokyo, Bunkyo, Tokyo 113-0033 Japan; 7 Present Address: Department of Engineering Science, University of Electro-Communications, Chofu, Tokyo 182-8585 Japan; 8 Present Address: Department of Physics, Osaka University, Toyonaka, Osaka 560-0043 Japan

**Keywords:** Condensed-matter physics, Superconducting properties and materials

## Abstract

One of the most significant issues for superconductivity is clarifying the momentum-dependent superconducting gap Δ(${\boldsymbol{k}}$), which is closely related to the pairing mechanism. To elucidate the gap structure, it is essential to investigate Δ(${\boldsymbol{k}}$) in as many different physical quantities as possible and to crosscheck the results obtained in different methods with each other. In this paper, we report a combinatorial investigation of the superfluid density and the flux-flow resistivity of iron-pnictide superconductors; LiFeAs and BaFe_2_(As_1−*x*_P_*x*_)_2_ (*x* = 0.3, 0.45). We evaluated Δ(${\boldsymbol{k}}$) by fitting these two-independent quantities with a two-band model simultaneously. The obtained Δ(${\boldsymbol{k}}$) are consistent with the results observed in angle-resolved photoemission spectroscopy (ARPES) and scanning-tunneling spectroscopy (STS) studies. We believe our approach is a powerful method for investigating Δ(${\boldsymbol{k}}$) because it does not require a sample with clean surface unlike ARPES and STS experiments, or a rotational magnetic-field system for direct measurements of the angular dependence of thermodynamic quantities.

## Introduction

Although conventional superconductors and cuprates possess definitive Δ(***k***) with $s$- and $d$-wave symmetry, iron pnictides have multifarious Δ(***k***) comprised of combinations of gaps with- and without zero points (nodes) reflecting their multiple-band nature. An effective method for investigating Δ(***k***) is to measure physical quantities sensitive to low-energy quasiparticle excitations. In this paper, we focus on two of such physical quantities. The first quantity is the temperature-dependent superfluid density ${n}_{s}(T)$ at $T\ll {T}_{{\rm{c}}}$ (${T}_{{\rm{c}}}$ is the superconducting-transition temperature). For a single-gap superconductor in the clean limit, ${n}_{s}(T)$ behaves as ${n}_{s}\mathrm{(0)}-{n}_{s}(T)\propto \exp [-\Delta (T)/{k}_{{\rm{B}}}T]$ when Δ(***k***) has no nodes. Meanwhile, a nodal-gap superconductor exhibits ${n}_{s}\mathrm{(0)}-{n}_{s}(T)\propto {(T/{T}_{{\rm{c}}})}^{\beta }$ with $\beta =1$ and 2 when Δ(***k***) contains line-like and point-like nodes, respectively. The situation becomes more complicated in multiple-gap cases because the characteristics of every gap contribute to ${n}_{s}(T)$. The second quantity addressed herein is the magnetic-field dependence of the flux-flow resistivity ${\rho }_{f}(B)$, which is a finite dissipation induced by quasiparticles bound inside the vortex core where $\Delta$ is suppressed locally^1,2^. ${\rho }_{f}(B\parallel c)$ behaves as ${\rho }_{f}(B)/{\rho }_{n}=\alpha B/{B}_{c2}$ in the $B\ll {B}_{{\rm{c}}2}$ region (${\rho }_{n}$ is the normal-state resistivity and ${B}_{{\rm{c}}2}$ is the upper critical field in the $B\parallel c$ configuration). The initial slope, $\alpha$, relates to Δ(***k***) through vortex-core-bound states and increases from unity with increasing anisotropy of Δ(***k***)^2–6^. Kopnin and Volovik^7^ successfully reproduced such an empirical relation between $\alpha$ and Δ(***k***) in single-gap superconductors as $\alpha =\langle {\Delta }_{0}^{2}\rangle /\langle {\Delta }^{2}({\boldsymbol{k}})\rangle$, where Δ_0_ is the maximum magnitude of superconducting gap and $\langle \cdots \rangle =\int \,{\rm{d}}{S}_{{\rm{F}}}(\cdots )/|\hslash {{\boldsymbol{v}}}_{{\rm{F}}}|$ is the Fermi surface average. Furthermore, systematic investigations on iron pnictides clarified that the Kopnin-Volovik relation also holds in multiple-gap superconductors at least in a qualitative manner^8–12^. These results encouraged us to investigate Δ(***k***) more quantitatively, based on ${n}_{s}(T)$ and ${\rho }_{f}(B)$ studies.

## Results and Discussion

### Superfluid density and flux-flow resistivity measurements

**Figure 1:**
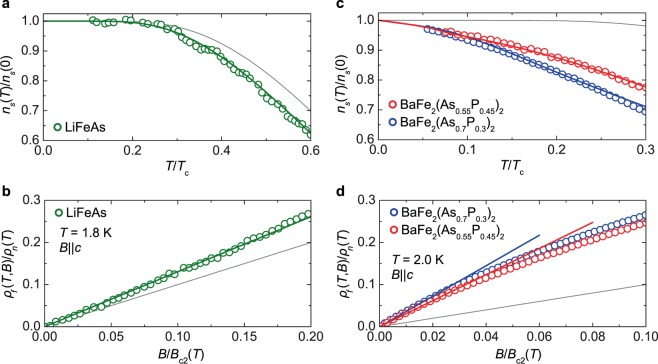
Experimental results. (**a**,**b**) $T$-dependence of the superfluid-density fraction in the zero-field limit (**a**) and $B$-dependence of the normalized flux-flow resistivity at 1.8 K($\simeq$0.1*T*_c_) (**b**) of LiFeAs. (**c**,**d**) The same plots as in (**a** and **b**) at 2 K($\simeq$0.07*T*_c_) for BaFe_2_(As_1−*x*_P_*x*_)_2_. The thick curves are the results of the two-band model (see the text), and the thin curves are the expected behavior for an isotropic gap with Δ(0)/${k}_{{\rm{B}}}{T}_{{\rm{c}}}=1.76$.

We investigated the superfluid density and flux-flow resistivity by measuring the microwave surface impedance in the zero-field limit or under finite magnetic field, respectively. Details of measurements and data analysis are presented in Supplementary Information. The superfluid-density fraction, ${n}_{s}(T)/{n}_{s}\mathrm{(0)}$, of LiFeAs saturated to unity below ≈0.25*T*_c_ (Fig. 1a). This behavior suggests that Δ(***k***) has no nodes, consistent with previous reports^8,13,14^. As for the flux-flow resistivity, ${\rho }_{f}(B)$ of LiFeAs increased with $\alpha$ moderately greater than unity (Fig. 1b), indicating that Δ(***k***) has a finite anisotropy^8^.

In contrast to that for LiFeAs, ${n}_{s}(T)/{n}_{s}\mathrm{(0)}$ for BaFe_2_(As_1−*x*_P_*x*_)_2_ (Fig. 1c) did not reach unity, even at $0.05{T}_{{\rm{c}}}$, and it exhibited quasi-linear $T$ dependence (${n}_{s}(T)/{n}_{s}(0)\approx 1-{T}^{\beta }$), suggesting the presence of line-nodal gaps. The exact values of $\beta$ obtained from the data below $0.3{T}_{{\rm{c}}}$ were $\beta =1.45\pm 0.05$ ($x=0.30$) and $1.65\pm 0.05$ ($x=0.45$)^12^ (see Supplementary Fig. S2). Possible origins of such fractional $\beta$ values are (i) a pair-breaking effect due to impurity scattering^15,16^, (ii) a renormalization of the effective Fermi velocity due to quantum fluctuations^17,18^, and (iii) multiple-band nature of the material^19^. The first two explanations are unlikely for BaFe_2_(As_1−*x*_P_*x*_)_2_ because the residual dc resistivity of our samples was small (see Supplementary Fig. S1) and fractional $\beta$ was also observed in BaFe_2_(As_0.55_P_0.45_)_2_^12^, which has a composition far from the quantum critical point ($x\approx 0.3$). Regarding the flux flow, the value of $\alpha$ for BaFe_2_(As_1−*x*_P_*x*_)_2_ was high, exceeding 3, which is greater than that observed in cuprates ($\alpha \approx 2$), whose gap is completely anisotropic^20^. Thus, a very large $\alpha$ suggests that the multiple-band effect plays an important role in addition to the highly anisotropic gap structure.

Summarizing the experimental results, it is expected that LiFeAs has nodeless gaps with moderate anisotropy and that BaFe_2_(As_1−*x*_P_*x*_)_2_ has highly anisotropic gaps containing at least one line-nodal gap. Furthermore, the multiple-band effect should play an essential role. To check these hypotheses concerning the Δ(***k***) of these materials, we evaluated Δ(***k***) quantitatively by fitting the ${n}_{s}(T)$ and ${\rho }_{f}(B)$ data with a phenomenological model, as described below.

### The model we used to fit the data

We considered two sheets of Fermi surface composed of one-hole and one-electron pockets as a minimum model. Such a two-band model has been used elsewhere and is justified when the phase of the wave function on each band does not play a crucial role. Hereafter, we use subscript “h” (“e”) to specify hole (electron)-like band component. Although unusual phenomena, such as a time-reversal-symmetry-breaking state originating from Josephson-type inter-band interactions among *N*(>3)-band components^21^, may affect on superfluid density and flux-flow resistivity, but considering such exotic contributions is beyond our purpose of this manuscript that to demonstrate a new methodology to evaluate anisotropy of superconducting gaps on multiple-band superconductors from experimentally obtained data. We hope that our attempt in this manuscript will stimulate and promote more sophisticated theoretical researches in the future.

As a model for superfluid density in the $ab$ plane, ${n}_{s}(T)$, we used two-band extension of the Chandrasekhar-Einzel model developed for a single-gap superconductors^16,22^. That is, the superfluid-density fraction of a two-band superconductor is given by ${n}_{s}(T)/{n}_{s}(0)=\gamma {n}_{s{\rm{h}}}(T)/{n}_{s{\rm{h}}}(0)+(1-\gamma ){n}_{s{\rm{e}}}(T)/{n}_{s{\rm{e}}}(0)$, where $\gamma$ is a weight factor determined by the Fermi-surface structure (see Section B in Supplementary Information). $\nu$(=h, e)-band component of superfluid density is

1 $${n}_{s\nu }(T)=\frac{{\mu }_{0}{e}^{2}}{4{\pi }^{3}\hslash }{\langle {({{\boldsymbol{v}}}_{{\rm{F}}\nu }^{ab})}^{2}\left[1-\frac{1}{2{k}_{{\rm{B}}}T}{\int }_{0}^{\infty }{\rm{d}}\xi\ {\rm{sech} }^{2}\left(\frac{\sqrt{{\xi }^{2}+{\Delta }_{\nu }^{2}(T,{\boldsymbol{k}})}}{2{k}_{{\rm{B}}}T}\right)\right]\rangle }_{\nu },$$

where ${\mu }_{0}$, ${{\boldsymbol{v}}}_{{\rm{F}}\nu }^{ab}$, and ${\langle \cdots \rangle }_{\nu }=\int \,{\rm{d}}{S}_{{\rm{F}}\nu }(\cdots )/|\hslash {{\boldsymbol{v}}}_{{\rm{F}}\nu }|$ are the permeability in vacuum, the in-plane Fermi velocity and the surface integral on the $\nu$th sheet of the Fermi surface. According to Eq. (1), ${n}_{s\nu }(T)$ obviously reflects momentum dependences of superconducting gaps and Fermi surfaces^22^.

Regarding the flux-flow resistivity, an explanation of ${\rho }_{f}(B)$ in two-band superconductors were attempted in frameworks of two-band extension of time-dependent Ginzburg-Landau (2band-tdGL) theory^23^ and that of a non-equilibrium version of Usadel (2band-Keldysh-Usadel) theory^24^. These calculations showed that various values of initial slope $\alpha$ can be obtained depending on ratio of diffusion constants on different bands and/or paring interactions. Unfortunately, situations considered in these reports, superconductors in the dirty limit at $T\simeq {T}_{{\rm{c}}}$ (2band-tdGL theory^23^) and superconductors in the dirty limit (2band-Keldysh-Usadel theory^24^), are far away from what we measured in this manuscript (fairly clean superconductors at $T\ll {T}_{{\rm{c}}}$). In addition, these theories are not applicable to anisotropic-gap cases since momentum dependence of gaps is smeared out due to strong impurity scattering in the dirty limit. Therefore, we could not adopt these results to our data. Although an extension of the Keldysh-Eilenberger theory^25^, which can treat clean single-band superconductors with anisotropic gap in whole temperature range, to two-band case may give an rigorous description for ${\rho }_{f}(B)$, such a calculation needs heavy analytical and numerical calculations. Hence it does not meet our purpose in this manuscript to demonstrate a new approach to evaluate the gap anisotropy in multiple-band superconductors from experimentally obtained superfluid density and flux-flow resistivity.

Instead of deriving a formula for ${\rho }_{f}(B)$ based on rigorous but complicated calculations, we extended a parallel-circuit model for isotropic gaps^26,27^ to anisotropic-gap cases by applying the Kopnin-Volovik relation^7^ to each band component. The Goryo-Matsukawa model assumes that the inter-band interaction works to lock the relative phase between two gaps on different bands depending on the sign of inter-band interaction, which leads the situation that two fractional vortices flow together with the same velocity^26–28^. Such a picture is correct as long as measurements are carried out at low temperature^26,27^ and small driving force^28^, and microwave measurements reported in this manuscript meet these conditions. At this time, it is clarified that the flux-flow conductivity ($\mathrm{1/}{\rho }_{f}$) in two-band superconductors is given by $\mathrm{1/}{\rho }_{f}(B)=\mathrm{1/}{\rho }_{f{\rm{h}}}(B)+\mathrm{1/}{\rho }_{f{\rm{e}}}(B)$ from the viewpoint of energy minimization. We applied the Kopnin-Volovik relation to $\nu$-band component in order to take gap anisotropy into account; ${\rho }_{f\nu }(B\parallel c)/{\rho }_{n\nu }={\alpha }_{\nu }B/{B}_{{\rm{c}}2\nu }$ with ${\alpha }_{\nu }(T)={\langle {\Delta }_{\nu 0}^{2}(T)\rangle }_{\nu }/{\langle {\Delta }_{\nu }^{2}(T,{\boldsymbol{k}})\rangle }_{\nu }$, where Δ_*ν*0_, ${\rho }_{n\nu }$, and ${B}_{{\rm{c}}2\nu }$ are $\nu$(=h, e)-band components of the maximum magnitude of superconducting gap, the normal-state resistivity, and the characteristic field (corresponds to the upper critical field with zero inter-band interaction) in the $B\parallel c$ configuration. Unfortunately, ${\rho }_{f}(B)$ over the whole $B$ range for arbitral Δ(***k***) cannot be obtained theoretically, even in single-band superconductors. Thus, at present, we calculate the initial slope of ${\rho }_{f}(B)$ in two-band superconductors; $\alpha ={\rm{d}}({\rho }_{f}/{\rho }_{n})/{\rm{d}}(B/{B}_{{\rm{c}}2})$. Obtained expression for ${\rho }_{f}(B)$ in two-band superconductors at $B\parallel c\ll {B}_{{\rm{c}}2}^{{\rm{\min }}}(T)$ is

2 $$\frac{{\rho }_{f}(B,T)}{{\rho }_{n}(T)}=\frac{{B}_{{\rm{c}}2}^{{\rm{\max }}}(T)\left(\frac{1}{{\rho }_{n{\rm{h}}}(T)}+\frac{1}{{\rho }_{n{\rm{e}}}(T)}\right)}{\frac{{B}_{{\rm{c}}2{\rm{h}}}(T)}{{\alpha }_{{\rm{h}}}(T){\rho }_{n{\rm{h}}}(T)}+\frac{{B}_{{\rm{c}}2{\rm{e}}}(T)}{{\alpha }_{{\rm{e}}}(T){\rho }_{n{\rm{e}}}(T)}}\frac{B}{{B}_{{\rm{c}}2}^{{\rm{\max }}}(T)}=\alpha (T)\frac{B}{{B}_{{\rm{c}}2}^{{\rm{\max }}}(T)},$$

where ${B}_{{\rm{c}}2}^{{\rm{\min }}/{\rm{\max }}}$ are the smaller/larger value of ${B}_{{\rm{c}}2{\rm{h}}}$ and ${B}_{{\rm{c}}2{\rm{e}}}$. Obtained $\alpha$ reflects momentum dependences of Fermi sheets and superconducting gaps through ${\alpha }_{\nu }$ and ratios ${\rho }_{n{\rm{h}}}/{\rho }_{n{\rm{e}}}$ and ${B}_{{\rm{c}}2{\rm{h}}}/{B}_{{\rm{c}}2{\rm{e}}}$. We found that Eq. (2) becomes equivalent to that calculated on the basis of the 2band-Keldysh-Usadel theory^24^ with zero inter-band interaction when we impose isotropic gaps in the dirty limit. This means that Eq. (2) is the simplest extension of ${\rho }_{f}(B)$ in two-band superconductors without inter-band interactions to anisotropic gaps. In other words, it is difficult to evaluate the strength of inter-band interaction by present model. We hope that more-rigorous evaluation of ${\rho }_{f}(B)$ will be made by a Keldysh-Eilenberger theory^25^ extended to two-band superconductors with anisotropic gaps in the future. More-detailed information relating to our model described above are given in the Section B of Supplementary Information.

**Figure 2:**
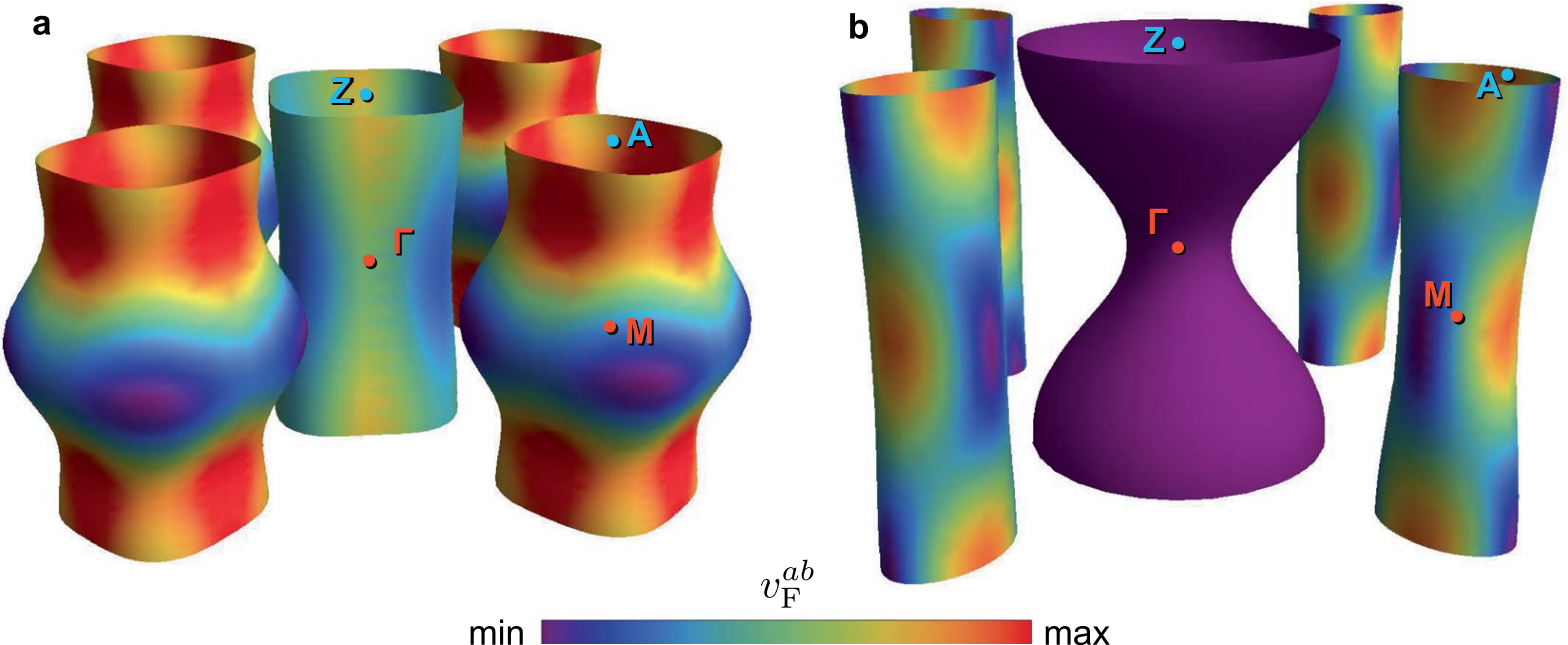
Schematics of the Fermi surface used for the two-band model analysis. (**a**,**b**) Sheets of the Fermi surface of LiFeAs (**a**) and of BaFe_2_(As_0.7_P_0.3_)_2_ (**b**), which we evaluated from ARPES data^29,30,34^. The coloring represents the relative magnitudes of the in-plane Fermi velocity, ${v}_{{\rm{F}}}^{ab}=|{{\boldsymbol{v}}}_{{\rm{F}}}^{ab}|$, of each materials. Since the substitution form As to P shorten the $c$-axis length, the Fermi surface of BaFe_2_(As_0.55_P_0.45_)_2_ should have ${k}_{z}$ dependence slightly larger than that of BaFe_2_(As_0.7_P_0.3_)_2_.

We assumed BCS-like $T$ dependence, Δ_*ν*_(*T*) = Δ_*ν*_ $(0)\tanh (1.785\sqrt{{T}_{{\rm{c}}}/T-1})$, instead of solving the gap equation self-consistently. Such a simplification is justified as long as we focus on the low-$T$ region because Δ$(T\ll {T}_{{\rm{c}}})$ is almost constant. Rather than using oversimplified Fermi cylinders, we used the exact ***k*** dependence of the Fermi surface that was evaluated from the ARPES data^29,30^ (Fig. 2). For superconductors for which ARPES measurement cannot be performed, the Fermi surface obtained by band calculations may be used as an alternative. As described above, ***k*** dependence of the Fermi surface is important since it is reflected on ${n}_{s}(T)$ and ${\rho }_{f}(B)$ through surface integrals on Fermi sheets. We set Δ_*ν*_(***k***) as

3 $${\Delta }_{\nu }({\boldsymbol{k}})=\left(\frac{{\Delta }_{\nu }^{{\rm{\max }}}+{\Delta }_{\nu }^{{\rm{\min }}}}{2}\right)+{p}_{\nu }\left(\frac{{\Delta }_{\nu }^{{\rm{\max }}}-{\Delta }_{\nu }^{{\rm{\min }}}}{2}\right)\,\cos \,({q}_{\nu }\varphi )\,\cos \,\left({r}_{\nu }\frac{{k}_{z}c}{2}\right),$$

where $\varphi$ is the azimuth angle measured from the Γ-M direction in the Brillouin zone (Fig. 2), and ${\Delta }_{\nu }^{{\rm{\max }}}$ (${\Delta }_{\nu }^{{\rm{\min }}}$) is the maximum (minimum) value of Δ_*ν*_(***k***). By considering the Fermi-surface symmetry and referring to the ARPES data^31^, we selected prefactors $({p}_{{\rm{h}}},{q}_{{\rm{h}}},{r}_{{\rm{h}}},{p}_{{\rm{e}}},{q}_{{\rm{e}}},{r}_{{\rm{e}}})$ of LiFeAs as $(\,-\,1,4,0,1,4,0)$, which leads to an in-plane four-fold anisotropy. In contrast, $(1,0,1,1,2,1)$ was used for BaFe_2_(As_1−*x*_P_*x*_)_2_, which has a possibility of appearance of horizontal-nodal lines in Δ_h_^30,32^ and/or loop-nodal lines in Δ_e_^33,34^. These prefactors are selected so that the superconducting gaps reflect the symmetry of hole- and electron-like Fermi surface. For example, in the case of BaFe_2_(As_1−*x*_P_*x*_)_2_, $({p}_{{\rm{h}}},{q}_{{\rm{h}}},{r}_{{\rm{h}}})=(1,0,1)$ expects an isotropy in the ${k}_{x}$-${k}_{y}$ plane and a two-fold symmetry in the ${k}_{z}$ direction but $({p}_{{\rm{e}}},{q}_{{\rm{e}}},{r}_{{\rm{e}}})=(1,2,1)$ gives two-hold symmetries in the ${k}_{x}$-${k}_{y}$ plane and along ${k}_{z}$ direction. These symmetry is the same to those of Fermi sheets of hole- and electron bands. Calculations with other prefactors are given in the Section C of Supplementary Information. Consequently, we can evaluate ${\Delta }_{\nu }^{{\rm{\max }}}$ and ${\Delta }_{\nu }^{{\rm{\min }}}$ as fit parameters through the simultaneous fitting of ${n}_{s}(T)$ and ${\rho }_{f}(B)$ with the model for the exact Fermi-surface structure.

### Fitted results

The results of the two-band model analysis are plotted as solid curves in Fig. 1, exhibiting good agreements with the measured data of ${n}_{s}(T\ll {T}_{{\rm{c}}})$ and ${\rho }_{f}(B\ll {B}_{{\rm{c}}2})$. The obtained fit parameters and its resolution are presented in Table 1 and Supplementary Table S1, respectively. The obtained gap anisotropies were reasonable compared to those measured by other probes, as mentioned below, validating our approach to Δ(***k***) determination.

**Table 1:** Superconducting gaps estimated by the two-band model analysis.

Material	${{\boldsymbol{\Delta }}}_{{\bf{h}}}^{{\bf{m}}{\bf{a}}{\bf{x}}}$	${{\boldsymbol{\Delta }}}_{{\bf{h}}}^{{\bf{m}}{\bf{i}}{\bf{n}}}$	${{\boldsymbol{\Delta }}}_{{\bf{e}}}^{{\bf{m}}{\bf{a}}{\bf{x}}}$	$|{{\boldsymbol{\Delta }}}_{{\bf{e}}}^{{\bf{m}}{\bf{i}}{\bf{n}}}|$	Probe
LiFeAs	_1.7 ± 0.2_	_1.1 ± 0.1_	_2.6 ± 0.3_	_1.8 ± 0.2_	
	_1.9 ± 0.2_	_1.4 ± 0.2_	_1.9 ± 0.1_	_1.8 ± 0.1_	ARPES^31^
	_1.9 ± 0.2_	_≈1.3_	_—_	_—_	STM^35^
BaFe_2_(As_0.7_P_0.3_)_2_	_3.1 ± 0.3_	_0.43 ± 0.1_	_1.1 ± 0.1_	_3.1 ± 0.3_	
	_3.1 ± 0.3_	_0 ± 0.3_	_3.5 ± 0.3_	_2.7 ± 0.3_	ARPES^30^
	_3.1 ± 0.4_	_1.9 ± 0.4_	_≈0.8_	_≈3.1_	ARPES^34^
BaFe_2_(As_0.55_P_0.45_)_2_	_3.7 ± 0.4_	_0.51 ± 0.1_	_1.7 ± 0.2_	_3.7 ± 0.4_	

Superconducting gaps reported by other probes are also listed for comparison. All values are in units of *k*_B_*T*_c_.

**Figure 3:**
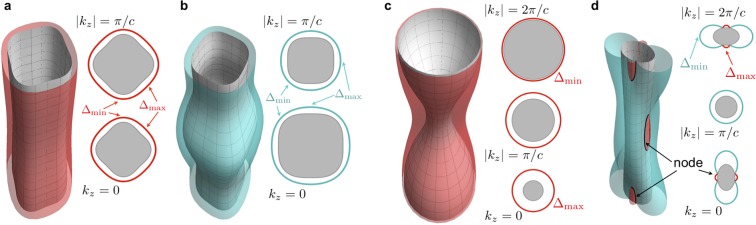
Fitted results of the superconducting gaps. Schematic images of Δ(***k***) on the Fermi surface for LiFeAs (**a**,**b**) and for BaFe_2_(As_1−*x*_P_*x*_)_2_ (**c**,**d**). The right-hand parts of each panels are cross-sectional slices at ${k}_{z}=0,\pi /c$, and $2\pi /c$. The gray-shaded areas correspond to the Fermi sea, and the distance from the Fermi surface represents the magnitude of Δ(***k***).

Δ_h_(***k***) and Δ_e_(***k***) of LiFeAs were finite over the entire hole- and electron-like sheets of the Fermi surface (Fig. 3a,b), and their minima were located in the Γ-M and M-M directions, respectively. A barometer of gap modulation defined by ${M}_{\nu }\equiv 1-{\Delta }_{\nu }^{{\rm{\min }}}/{\Delta }_{\nu }^{{\rm{\max }}}$ was ${M}_{{\rm{h}}}\approx 36 \%$ and ${M}_{{\rm{e}}}\approx 29 \%$. Such moderately anisotropic gaps and these ***k*** dependences are consistent with those reported in refs. ^31,35^.

Next, we consider BaFe_2_(As_1−*x*_P_*x*_)_2_ with $x=0.3$. The results for $x=0.45$ was similar to the case of $x=0.3$ qualitatively. Δ_h_ of BaFe_2_(As_0.7_P_0.3_)_2_ is finite over the entire hole-like sheet similar to LiFeAs, but its anisotropy was remarkably high up to ${M}_{{\rm{h}}}\approx \mathrm{87 \% }$ (Fig. 3c). ${\Delta }_{{\rm{h}}}^{{\rm{\min }}}$ appeared around point X, where horizontal nodes were reported via ARPES measurement^30^, and its magnitude (≈$1\,{\rm{meV}}$) was smaller than the energy resolution of ref. ^30^. Thus, the Δ_h_ based on our analysis is consistent with that of ref. ^30^ if we assume that the horizontal nodes in Δ_h_ reported in ref. ^30^ are actually small minima. Regarding the electron-like band, our result suggested that Δ_e_ has loop-like nodes at the flat parts of the Fermi surface (Fig. 3d). The emergence of loop nodes is consistent with results of the angle-resolved thermal-conductivity study^33^ and the ARPES study^34^. In particular, note that the location where the loop nodes appear is almost the same as that suggested in ref. ^33^.

Δ_*ν*_ between our calculations and results of other experiments have slight difference in a quantitative manner, and there are two possibilities for this difference; the first one is the difference in sample properties and second origin is additional effects caused by inter-band coupling not included in our model. LiFeAs is sensitive to air and impurities, and characteristics in BaFe_2_(As_1−*x*_P_*x*_)_2_ are sensitive to phosphorus contents. Slight difference in such sample conditions might affect on the value of superconducting gaps. As for the second possibility, our model is the simplest extension of two-band superconductors in the zero inter-band interaction limit to anisotropic-gaps cases. Exotic phenomena, such as the time-reversal-symmetry-breaking state expected in *N*(>3)-band superconductors^21^, and/or non-trivial effects originating from multiple-band components, which were not included in our model, may influence on actual ${n}_{s}(T)$ and ${\rho }_{f}(B)$. We hope that further developments of our model will give more precise description of ${n}_{s}(T)$ and ${\rho }_{f}(B)$ in multiple-band superconductors and allow for more accurate evaluation of gap anisotropies in the future.

Finally, we refer to the comparison between BaFe_2_(As_0.7_P_0.3_)_2_ and BaFe_2_(As_0.55_P_0.45_)_2_, where $\alpha$ were found to be almost the same but the ${n}_{s}(T)$ values clearly differed. Examining the formulae of the two-band model (Section B in Supplementary Information), ${n}_{s}(T\ll {T}_{{\rm{c}}})$ is expected to be sensitive to smaller parts of Δ(***k***) while the flux-flow resistivity reflects the square of Δ(***k***) averaged over the Fermi surface. Obtained parameters listed in Table 1 show that ${\Delta }_{{\rm{h}}}^{{\rm{\min }}}/{k}_{{\rm{B}}}{T}_{{\rm{c}}}$ and $|{\rm{d}}{\Delta }_{{\rm{e}}}({\boldsymbol{k}})/{\rm{d}}{\boldsymbol{k}}{|}_{{\boldsymbol{k}}\to {{\boldsymbol{k}}}_{{\rm{F}}}}$ (the slope of Δ_e_(***k***) approaching to gap nodes at the Fermi surface) of BaFe_2_(As_0.55_P_0.45_)_2_ were larger than those of BaFe_2_(As_0.7_P_0.3_)_2_. These values are consistent with the fact that ${n}_{s}(T\ll {T}_{{\rm{c}}})$ of BaFe_2_(As_0.55_P_0.45_)_2_ changed slowly in comparison with that of BaFe_2_(As_0.7_P_0.3_)_2_. On the other hand, ${M}_{{\rm{h}},{\rm{e}}}$ of BaFe_2_(As_0.7_P_0.3_)_2_ and BaFe_2_(As_0.55_P_0.45_)_2_ were close to each other and differences in anisotropy of the Fermi surface between these two compounds are not so remarkable. These characteristics lead each of ${\alpha }_{{\rm{h}}}$ and ${\alpha }_{{\rm{e}}}$ of BaFe_2_(As_0.7_P_0.3_)_2_ and BaFe_2_(As_0.55_P_0.45_)_2_ to be similar values. Therefore, observed ${n}_{s}(T\ll {T}_{{\rm{c}}})$ and ${\rho }_{f}(B\ll {B}_{{\rm{c}}2})$ can be understood by the difference in sensitivity of superconducting-gap structure; ${n}_{s}(T\ll {T}_{{\rm{c}}})$ is sensitive to smaller parts of Δ(***k***) and ${\rho }_{f}(B\ll {B}_{{\rm{c}}2})$ is sensitive to anisotropy of Δ(***k***). In other words, these results suggest that we can evaluate Δ(***k***) from two independent physical quantities of ${n}_{s}(T\ll {T}_{{\rm{c}}})$ and ${\rho }_{f}(B\ll {B}_{{\rm{c}}2})$ by using different gap sensitivities.

## Conclusion

We measured ${n}_{s}(T)$ and ${\rho }_{f}(B)$ by using a microwave technique and fitted the data with a phenomenological model developed for two-band systems that considered the Fermi-surface structure. As a result, we found that LiFeAs has nodeless gaps with moderate anisotropy. In contrast, the data for BaFe_2_(As_1−*x*_P_*x*_)_2_ ($x=0.3,0.45$) can be reproduced by a highly anisotropic nodeless gap on the hole-like sheet and another gap with loop-like nodes on the electron-like sheet. These results are consistent with those for Δ(***k***) obtained using other probes and reasonable in quantitatively, thereby validating our combinatorial investigation of ${n}_{s}(T)$ and ${\rho }_{f}(B)$.

Our approach has several advantages over other probes for investigating Δ(***k***); neither ${n}_{s}(T)$ nor ${\rho }_{f}(B)$ measurements require (i) a clean- and uncharged sample surface, unlike ARPES and STS investigations, and (ii) a rotational magnetic-field system for angle-resolved measurements of thermodynamic quantities. Furthermore, these measurements can be performed in a lower-$T$ region than the typical ARPES measurement, meaning that our approach can be applied to a broader range of superconductors. Thus, we believe that our approach is a novel and powerful method for investigating superconducting-gap structures.

As for further advance of our model, it is expected that an extension of the Keldysh-Eilenberger theory^25^ to multiple-band superconductors with anisotropic gaps including effects caused by inter-band interactions will give more precise expression of flux-flow resistivity in multiple-band superconductors. We hope that our approach for gap-anisotropy evaluation based on our phenomenological two-band model for ${n}_{s}(T)$ and ${\rho }_{f}(B)$ stimulates more sophisticated theoretical studies on it in the future.

## Methods

Single crystals of LiFeAs (${T}_{{\rm{c}}}=18\,{\rm{K}}$), BaFe_2_(As_0.7_P_0.3_)_2_ (${T}_{{\rm{c}}}=29.5\,{\rm{K}}$), and BaFe_2_(As_0.55_P_0.45_)_2_ (${T}_{{\rm{c}}}=22.5\,{\rm{K}}$) were synthesized by self-flux methods^36,37^. These samples exhibited the residual dc resistivity of less than 35 *μ*Ωcm (see Supplementary Fig. S1), evidencing the high quality of the samples. ${n}_{s}(T)$ and ${\rho }_{f}(B)$ were obtained from microwave surface impedance measured by using a cavity perturbation technique in the zero-field limit and under finite magnetic fields of up to 8 T, respectively. Detailed information on these procedures is presented in the Section A of Supplementary Information.

## Supplementary information

Supplementary Information.
