# Supplementary material for: Superconducting-Gap Anisotropy of Iron Pnictides Investigated via Combinatorial Microwave Measurements
Source: Sci Rep. 2020 Apr 27;10:7064. doi: 10.1038/s41598-020-63304-0 (PMC7184760; doi:10.1038/s41598-020-63304-0)
Supplement: Supplementary file 1 — Supplementary Information. [file 41598_2020_63304_MOESM1_ESM.pdf]

Supplementary Information for "Superconducting-Gap Anisotropy of Iron Pnictides Investigated via Combinatorial Microwave Measurements"

Tatsunori Okada[1,2,*], Yoshinori Imai[1,3], Kentaro Kitagawa[4,†], Kazuyuki Matsubayashi[4,‡], Masamichi Nakajima[5,§], Akira Iyo[5], Yoshiya Uwatoko[4], Hiroshi Eisaki[5], and Atsutaka Maeda[1,*]

[1]Department of Basic Science, University of Tokyo, Meguro, Tokyo, 153-8902, Japan
[2]Institute for Materials Research, Tohoku University, Sendai, Miyagi, 980-8577, Japan
[3]Department of Physics, Tohoku University, Sendai, Miyagi, 980-8578, Japan
[4]Institute for Solid State Physics, University of Tokyo, Kashiwa, Chiba, 277-8581, Japan
[5]National Institute of Advanced Industrial Science and Technology, Tsukuba, Ibaraki, 305-8568, Japan
[†]Present address: Department of Physics, University of Tokyo, Bunkyo, Tokyo, 113-0033, Japan
[‡]Present address: Department of Engineering Science, University of Electro-Communications, Chofu, Tokyo, 182-8585, Japan
[§]Present address: Department of Physics, Osaka University, Toyonaka, Osaka, 560-0043, Japan
[*]Correspondence and requests for materials should be addressed to T.O. (e-mail: tatsu.okada@imr.tohoku.ac.jp) or A.M. (e-mail: cmaeda@mail.ecc.u-tokyo.ac.jp)

ABSTRACT

This manuscript contains supplementary information on experiments and model calculations reported in the manuscript entitled "Superconducting-Gap Anisotropy of Iron Pnictides Investigated via Combinatorial Microwave Measurements".

A) Detailed information of experiments

Cavity perturbation technique

As described below, the superfluid density and the flux-flow resistivity were obtained from the surface impedance $Z_s = R_s - iX_s$. We used a cavity perturbation technique to measure Z_s. This technique is a unique method for investigating flux-flow phenomena in high-T_c superconductors, in which measurement in the flux-flow state via throwing dc current is impossible because of the strong vortex pinning. We used two cylindrical cavity resonators, which were composed of oxygen-free Cu and operated in the TE_{011} mode (Fig.S1**a**). The resonant frequency and the quality factor of the resonators were $(\omega/2\pi, Q) \approx (19\ \mathrm{GHz}, 6\times10^4)$ and $(44\ \mathrm{GHz} 2.6\times10^4)$, respectively. Both the external magnetic field, $B_{dc} \leq 8$ T, and the microwave magnetic field, B_ω, were applied parallel to the c axis of the sample (Fig.S1**b**). Thus, we investigated the in-plane vortex motion.

The surface resistance, R_s, and the surface reactance, X_s, are related to ω and Q measured with the sample (denoted with the subscript "sample") and without the sample ("blank") as

$$R_s = G\left(\frac{1}{2Q_{\mathrm{sample}}} - \frac{1}{2Q_{\mathrm{blank}}}\right), \quad X_s = G\left(1 - \frac{\omega_{\mathrm{sample}}}{\omega_{\mathrm{blank}}}\right) + C, \qquad \text{(S1)}$$

where G and C are constants determined by the geometry of the sample and the resonator. We assumed the Hagen-Rubens relation, $R_s = X_s = \sqrt{\mu_0\omega\rho_{dc}/2}$, in the normal state to determine G and C. μ_0 is the vacuum permeability, and ρ_{dc} is the dc resistivity of the sample measured by using a four-probe method (Fig.S1**c**). For an example, we plotted $Z_s(B,T)$ obtained by this technique for $BaFe_2(As_{0.7}P_{0.3})_2$ in Fig.S1**d**. The good agreement of the data measured using two different operations, namely, B-sweep measurements (solid curves) and T-sweep measurements (open circles), indicates that magnetic vortices exist in the sample uniformly, at least in the effective region where the magnetic field penetrates.

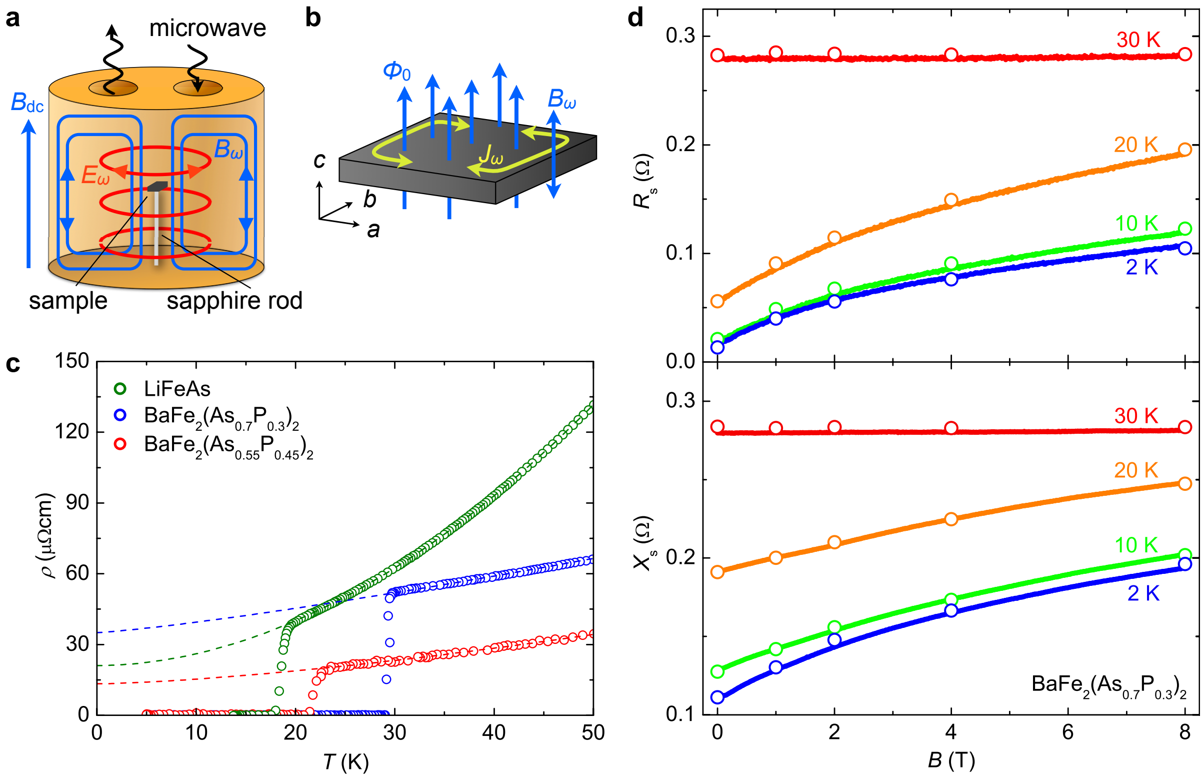

Supplementary Figure S1. Experimental setup. a, Schematic image of the cavity resonator. A sample was mounted onto the sapphire rod to position the sample at the center of the resonator, where microwave magnetic field, B_ω, is strongest. External dc magnetic fields, B_{dc}, up to 8 T were applied parallel to the c axis of the sample by a superconducting-solenoid magnet. **b**, Enlarged image of the sample. The sample was cut into dimensions of $a \times b \times c \approx 0.5 \times 0.5 \times 0.2\ \mathrm{mm}^3$. **c**, T-dependent dc resistivity, $\rho_{dc}(T)$. The dashed curves are the normal-state resistivity, $\rho_n(T)$, estimated by extrapolating $\rho_{dc}(T > T_c)$ to the superconducting state. **d**, $Z_s(B, T, \omega/2\pi = 44\ \mathrm{GHz})$ of $BaFe_2(As_{0.7}P_{0.3})_2$. The solid curves and open circles are the data measured by sweeping B at fixed T and by sweeping T under fixed B, respectively.

Superfluid density and flux-flow resistivity

The T-dependent superfluid density, $n_s(T) = 1/\lambda^2(T)$ (λ is the magnetic penetration depth), can be obtained from Z_s measured in the zero-field limit ($B_{dc} = 0$) via the relation $\lambda(T) = X_s(T)/\mu_0\omega$ (Fig.S2**a**). As clearly seen in Fig.S2**b**, $n_s(T)$ of $BaFe_2(As_{1-x}P_x)_2$ changed with temperature, with fractional β being between 1 and 2. These results are different from the conventional values for the line-nodal gap ($\beta = 1$) and for the gapless state due to impurity scattering ($\beta = 2$). We attributed such fractional β to the multiple-band nature of these materials[1], and our analysis based on two-band model supports this idea (see the main text).

The B-dependence of the flux-flow resistivity was obtained from the data measured under finite fields ($B_{dc} > 0$) using the Coffey-Clem model[2]. In this model, Z_s induced by the vortex motion is calculated considering the effects of normal fluids and of the flux creep due to thermal fluctuations. Because these effects are negligibly small at sufficiently low temperatures, Z_s can be described as

$$Z_s = -i\mu_0\omega\lambda\sqrt{1 + i\frac{\rho_f}{\mu_0\omega\lambda^2}\frac{(\omega/\omega_{cr})^2 - i(\omega/\omega_{cr})}{1+(\omega/\omega_{cr})^2}}, \quad \text{(S2)}$$

where ω_{cr} characterizes the crossover from the reactive response ($\omega \ll \omega_{cr}$) to the resistive response ($\omega \gg \omega_{cr}$). Although the free-flux-flow state, where vortices move without suffering from pinning, is achieved only at higher frequencies (typically $\omega > 5\omega_{cr}$), we can obtain exact $\rho_f(B,T)$ due to the simultaneous measurement of R_s and X_s and equation (S2). The flux-flow resistivity of LiFeAs and $BaFe_2(As_{1-x}P_x)_2$ ($x = 0.3, 0.45$) measured at $T/T_c \approx 0.1$ are plotted in Fig.S2**c**. Enlarged plots of these data are presented in the main text as Fig.1.

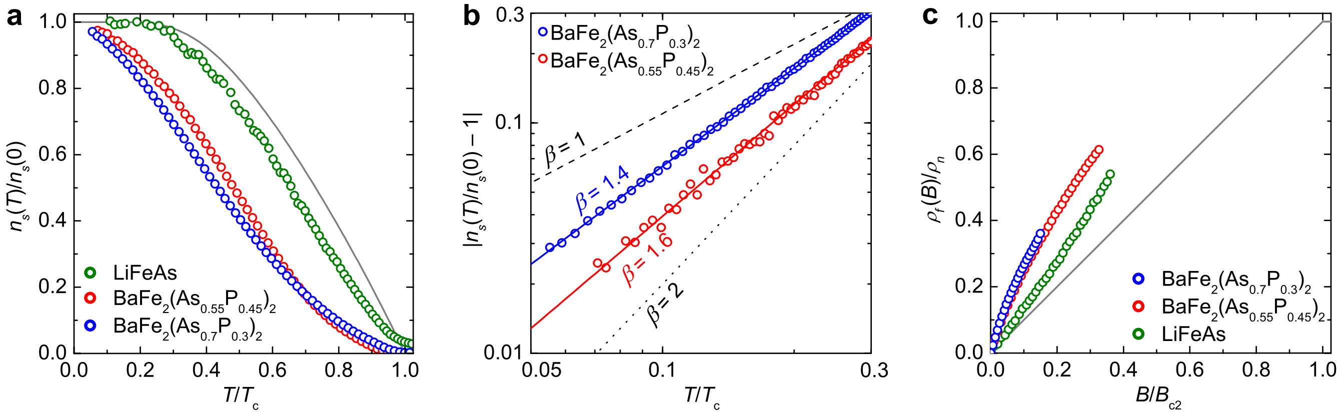

Supplementary Figure S2. Measured data. a, The superfluid-density fraction, $n_s(T)/n_s(T)$, of LiFeAs and $BaFe_2(As_{1-x}P_x)_2$ ($x = 0.3, 0.45$) throughout the superconducting phase. **b**, A double-logarithmic plot of $|n_s(T)/n_s(0) - 1|$ of $BaFe_2(As_{1-x}P_x)_2$ below $0.3T_c$. The lines are guides to the eye of $n_s(T) = n_s(0) - A(T/T_c)^\beta$ for several β. **c**, The B-dependent flux-flow resistivity, $\rho_f(B)$, of LiFeAs and $BaFe_2(As_{1-x}P_x)_2$ measured at 1.8 K and 2.0 K, respectively.

B) Formulae of the two-band model

For the superfluid-density fraction

As a minimum model, the superfluid density of N-band superconductors is expected to be the simple summation of each band contributions, $n_s(T) = \sum_{\nu=1}^{N} n_{s\nu}(T)$. According to the model suggested by Chandrasekhar and Einzel[3], the νth band component of the in-plane superfluid density, $n_{s\nu}(T)$, can be described by the in-plane Fermi velocity, $\boldsymbol{v}_{F\nu}^{ab}$, and the kinetic energy of the quasiparticles measured from the Fermi level, ξ, as

$$n_{s\nu}(T) = \frac{\mu_0 e^2}{4\pi^3\hbar}\left\langle (v_{F\nu}^{ab})^2\left[1 - \frac{1}{2k_B T}\int_0^\infty d\xi\ \mathrm{sech}^2\left(\frac{\sqrt{\xi^2 + \Delta_\nu^2(T,\boldsymbol{k})}}{2k_B T}\right)\right]\right\rangle_\nu, \quad \text{(S3)}$$

where $(v_{F\nu}^{ab})^2 = |\boldsymbol{v}_{F\nu}^{ab}|^2$ and $\langle\cdots\rangle_\nu = \int dS_{F\nu}(\cdots)/|\hbar\boldsymbol{v}_{F\nu}|$ is the surface integral of X on the νth sheet of the Fermi surface. The first term of the right-hand side of equation (S3) corresponds to $n_{s\nu}(0)$ because the second term vanishes at $T = 0$ K. These formulae give the superfluid-density fraction of N-band superconductors. Thus, the total superfluid-density fraction is given

as

$$\frac{n_s(T)}{n_s(0)} = \sum_{\nu=1}^{N} \gamma_\nu \frac{n_{s\nu}(T)}{n_{s\nu}(0)} = 1 - \sum_{\nu=1}^{N} \gamma_\nu \frac{\left\langle (v_{\mathrm{F}\nu}^{ab})^2 \int_0^\infty \mathrm{d}\xi \ \mathrm{sech}^2 \left(\frac{\sqrt{\xi^2 + \Delta_\nu^2(T,\boldsymbol{k})}}{2k_\mathrm{B}T} \right) \right\rangle_\nu}{2k_\mathrm{B}T \left\langle (v_{\mathrm{F}\nu}^{ab})^2 \right\rangle_\nu}, \tag{S4}$$

where $\gamma_\nu = n_{s\nu}(0) \big/ \sum_{\nu=1}^{N} n_{s\nu}(0) = \left\langle (v_{\mathrm{F}\nu}^{ab})^2 \right\rangle_\nu \Big/ \sum_{\nu=1}^{N} \left\langle (v_{\mathrm{F}\nu}^{ab})^2 \right\rangle_\nu$ is the weighting factor of the νth-band ingredient. The set of weighting factors satisfies $\sum_{\nu=1}^{N} \gamma_\nu = 1$. This formula can account for the $\boldsymbol{k}$ dependence of the Fermi surface and the superconducting gaps through the Fermi surface integral of $\boldsymbol{v}_\mathrm{F}$ and $\Delta_\nu^2(\boldsymbol{k})$. In the manuscript, we used $N = 2$ and considered hole-like and electron-like sheets of the Fermi surface.

For the flux-flow resistivity

Recently, the magnetic field dependence of flux-flow resistivity, $\rho_f(B)$, in two-band superconductors has been studied on the basis of two-band version of the Keldysh-Usadel equation[4] and time-dependent Ginzburg-Landau (tdGL) equations[5]. These studies show that various kinds of initial slope, $\alpha = \mathrm{d}(\rho_f/\rho_n)/\mathrm{d}(B/B_{c2})$, reflecting the difference in diffusion constant on each bands, $D_\nu = v_{\mathrm{F}\nu}^2 \tau_\nu/3$, can be obtained, suggesting the importance of multiple-band effects. Unfortunately, these reports assumed two isotropic gaps and considered situation is far from that in our measurement (fairy clean superconductors, $T \ll T_\mathrm{c}$) since the Keldysh-Usadel equation is applicable to dirty superconductors and the tdGL equation is adequate for limited regions in the B-T phase diagram where superconducting order parameter is suppressed. An extension of a non-equilibrium quasi-classical (Keldysh-Eilenberger) theory[6], which can treat clean superconductors, to multiple bands with anisotropic gaps may give rigorous description for $\rho_f(B)$ in multiple-band system. However, it needs heavy numerical calculations, and hence it does not meet the purpose of this manuscript to demonstrate a new approach to evaluate the anisotropy of superconducting gaps in multiple-band materials from measured superfluid density and flux-flow resistivity by a microwave technique. Instead of such a rigorous calculation based on microscopic framework, we used the Goryo-Matsukawa model[7,8], which is the simplest application of the Bardeen-Stephen model to two-band systems and succeeds in explaining $\rho_f(B)$ in MgB_2[9] and Y_2C_3[10], as a minimal model to describe the flux-flow resistivity in two-band superconductors.

The Ginzburg-Landau free energy for two-band superconductors[7,8,11,12] is given by

$$F_\mathrm{S} = F_\mathrm{N} + \int \mathrm{d}^3\boldsymbol{r} \left\{ \sum_{\nu=1}^{2} \left[a_\nu(T)|\Delta_\nu|^2 + \frac{b_\nu}{2}|\Delta_\nu|^4 + \sum_{j=x}^{z} k_{\nu j} \left| \left(\frac{\partial}{\partial x_j} - \mathrm{i}\frac{e^* A_j}{\hbar} \right) \Delta_\nu \right|^2 + \sum_{\mu \neq \nu}^{2} g_{\mu\nu} \Delta_\mu \Delta_\nu^* \right] + \frac{|\nabla \times \boldsymbol{A}|^2}{2\mu_0} \right\}, \tag{S5}$$

where $\boldsymbol{A}(\boldsymbol{r})$ is a vector potential. The fourth term is a Josephson-type interaction $g_{12}\left(\Delta_1\Delta_2^* + \Delta_1^*\Delta_2\right) = g_{12}|\Delta_1||\Delta_2|\cos(\theta_1 - \theta_2)$, where θ_ν is a phase of superconducting wave function of νth-band component and $g_{12} = g_{21}$ is assumed. Regarding the inter-band Josephson coupling, dislocation of one magnetic vortex into two vortices with fractional flux quanta due to large driving force[13] or entropic term at high temperatures[14] has been suggested theoretically. However, the dislocation of fractional vortices is difficult to occur in our case since the current (and the driving force) introduced by the microwave perturbation technique is sufficiently small and we carried out flux-flow measurements at low temperatures around 2 K. Thus, the inter-band Josephson interaction is expected to result in a locking effect of phase difference $\theta_1 - \theta_2 = 0$ ($g_{12} < 0$) or π ($g_{12} > 0$)[7,8,13,14]. This is the situation considered in Goryo-Matsukawa[7,8] and small driving force regions in Lin-Bulaevskii[13], and two fractional vortices flow with the same velocity. In this case, current applied to the superconductor is shared by two bands, $\boldsymbol{J} = \boldsymbol{J}_1 + \boldsymbol{J}_2$, and energy dissipations caused by flux motion[15] in the unit volume become $W = \rho_{f1}\boldsymbol{J}_1^2 + \rho_{f2}\boldsymbol{J}_2^2$. These expression are the same to that in currents flowing through a parallel circuit of two resistors; the net flux-flow resistivity is given by

$$\frac{1}{\rho_f(B,T)} = \frac{1}{\rho_{f1}(B,T)} + \frac{1}{\rho_{f2}(B,T)}. \tag{S6}$$

$\rho_{f\nu}(B,T)$ reaches the normal-state resistivity, $\rho_{n\nu}(B,T)$, when the applied field equals to "the upper critical field" of νth band in the zero inter-band coupling limit, $B_{c2\nu}(T)$[7,8]. Hence, $\rho_f(B,T)$ equals to the net normal-state resistivity at the highest upper critical field; $1/\rho_f(B_{c2}^{\max},T) = 1/\rho_n(T) \equiv 1/\rho_{n1}(T) + 1/\rho_{n2}(T)$ with $B_{c2}^{\max}(T) \equiv \max\{B_{c21}(T), B_{c22}(T)\}$. In the zero inter-band interaction case, coefficients in the GL free energy for two-band superconductors (equation (S5)) in the clean limit become

$$a_\nu(T) \simeq N_\nu(E_\mathrm{F})\left(\frac{T}{T_{c\nu}} - 1\right), \quad b_\nu = \frac{7\zeta(3)N_\nu(E_\mathrm{F})}{8(\pi k_\mathrm{B} T_{c\nu})^2} \frac{\left\langle \Delta_\nu^4(\boldsymbol{k}) \right\rangle_\nu}{\left\langle \Delta_\nu^2(\boldsymbol{k}) \right\rangle_\nu^2}, \quad \tilde{k}_{\nu j} = \frac{\hbar^2 N_\nu(E_\mathrm{F})}{4(\pi k_\mathrm{B} T_{c\nu})^2} \frac{\left\langle (\boldsymbol{v}_{\mathrm{F}\nu} \otimes \boldsymbol{v}_{\mathrm{F}\nu})_{jj} \Delta_\nu^2(\boldsymbol{k}) \right\rangle_\nu}{\left\langle \Delta_\nu^2(\boldsymbol{k}) \right\rangle_\nu}, \quad g_{\mu\nu} = 0, \tag{S7}$$

where $T_{c\nu} \propto \Delta_{\nu 0}$, $N_\nu(E_\mathrm{F})$, and $\boldsymbol{v}_{\mathrm{F}\nu} \otimes \boldsymbol{v}_{\mathrm{F}\nu}$ are νth-band component of critical temperature, density of states at the Fermi energy,

and a tensor product of Fermi velocity. Consequently, the νth-band component of in-plane upper critical field in the zero inter-band interaction limit is

$$B_{\mathrm{c2}\nu}(T)=\frac{\Phi_0}{2\pi}\left(\frac{k_\nu}{-a_\nu(T)}\right)^{-1}\simeq\frac{(k_{\mathrm{B}}T_{\mathrm{c}\nu})^2}{e^*\hbar}\frac{\left\langle\Delta_\nu^2(\boldsymbol{k})\right\rangle_\nu}{\left\langle\left|\boldsymbol{v}_{\mathrm{F}\nu}^{ab}\right|^2\Delta_\nu^2(\boldsymbol{k})\right\rangle_\nu}\left(1-\frac{T}{T_{\mathrm{c}\nu}}\right). \tag{S8}$$

In the case of single-band superconductors, the flux-flow resistivity behaves as $\rho_f(B,T)/\rho_n(B,T)=\alpha(T)B/B_{\mathrm{c2}}(T)$ at $B\ll B_{\mathrm{c2}}(T)$[15], and the slope $\alpha(T)$ in the case of anisotropic gap can be described by the Kopnin-Volovik relation[16]; $\alpha(T)=\left\langle\Delta_0^2(T)\right\rangle/\left\langle\Delta^2(\boldsymbol{k},T)\right\rangle$. Unfortunately, $\rho_f(0\ll B\lesssim B_{\mathrm{c2}})$ in fairly clean superconductors with an anisotropic gap is not understood even theoretically. To address the $\boldsymbol{k}$-dependent superconducting gaps in two-band superconductors, we applied the Kopnin-Volovik relation to each band component; $\rho_{f\nu}(B,T)/\rho_{n\nu}(B,T)=\alpha_\nu(T)B/B_{\mathrm{c2}\nu}(T)$ at $B\ll B_{\mathrm{c2}\nu}(T)$ with $\alpha_\nu(T)=\left\langle\Delta_{\nu0}^2(T)\right\rangle_\nu/\left\langle\Delta_\nu^2(\boldsymbol{k},T)\right\rangle_\nu$. As a result, we can evaluate the initial slope in two-band superconductors, α, as

$$\frac{\rho_f(B\ll B_{\mathrm{c2}}^{\min}(T))}{\rho_n(B,T)}=\frac{\dfrac{1}{\rho_{n1}(B,T)}+\dfrac{1}{\rho_{n2}(B,T)}}{\dfrac{B_{\mathrm{c21}}(T)}{\alpha_1(T)\rho_{n1}(B,T)B}+\dfrac{B_{\mathrm{c22}}(T)}{\alpha_2(T)\rho_{n2}(B,T)B}}=\frac{B_{\mathrm{c2}}^{\max}(T)\left(\dfrac{1}{\rho_{n1}(B,T)}+\dfrac{1}{\rho_{n2}(B,T)}\right)}{\dfrac{B_{\mathrm{c21}}(T)}{\alpha_1(T)\rho_{n1}(B,T)}+\dfrac{B_{\mathrm{c22}}(T)}{\alpha_2(T)\rho_{n2}(B,T)}}\frac{B}{B_{\mathrm{c2}}^{\max}(T)}, \tag{S9}$$

where $B_{\mathrm{c2}}^{\min}(T)=\min\{B_{\mathrm{c21}}(T),B_{\mathrm{c22}}(T)\}$. By considering that the scattering rate τ^{-1} is proportional to the density of states at the Fermi level $N(E_{\mathrm{F}})$, the normal-state conductivity can be approximated as $1/\rho_{n\nu}=V^{-1}\sum_{\boldsymbol{k}}2e^2(\boldsymbol{v}\otimes\boldsymbol{v})\tau(-\partial f(E)/\partial E)\propto\langle\boldsymbol{v}_{\mathrm{F}}\otimes\boldsymbol{v}_{\mathrm{F}}/N(E_{\mathrm{F}})\rangle_\nu$, where V is a volume and $f(E)$ is the Fermi distribution function.

Let us consider isotropic gaps ($\alpha_1=\alpha_2=1$) in the dirty limit, the initial slope of $\rho_f(B)$ in equation (S9) can be transformed as

$$\alpha(T)=\max\left\{\frac{T_{\mathrm{c1}}}{D_1},\frac{T_{\mathrm{c2}}}{D_2}\right\}\frac{N_1(E_{\mathrm{F}})D_1(T)+N_2(E_{\mathrm{F}})D_2(T)}{N_1(E_{\mathrm{F}})T_{\mathrm{c1}}(T)+N_2(E_{\mathrm{F}})T_{\mathrm{c2}}} \tag{S10}$$

by using $B_{\mathrm{c2}\nu}\propto T_{\mathrm{c}\nu}/D_\nu$ and $1/\rho_{n\nu}\propto N_\nu(E_{\mathrm{F}})D_\nu$ ($D_\nu=v_{\mathrm{F}\nu}^2\tau_\nu/3$ is the diffusion constant of νth band). This expression matches to equation (55) in Silaev-Vargunin[4], where $\rho_f(B)$ in two-band superconductors with $V_{12}=V_{21}=0$ in the dirty limit was calculated on the basis of the Keldysh-Usadel thepry, with small difference in factor of $1/\beta_0=1/0.9$[17]. Thus, equation (S9) is the simplest extension of $\rho_f(B)$ in two-band materials with zero inter-band interaction to anisotropic gaps.

In summary, we can calculate the initial slope α by taking the $\boldsymbol{k}$ dependence of the superconducting gap and the Fermi surface into account. We would like to note about further extension of our model given above. In cases of $N(\geq 3)$-band superconductor, it has been proposed that an emergence of time-reversal symmetry-broken state caused by the cross term, $\sum_{\nu=1}^{N}\sum_{\mu\neq\nu}^{N}g_{\mu\nu}\Delta_\nu\Delta_\mu^*$ (e.g. Silaev-Babaev[18]). Such a unusual state may affects $\rho_f(B)$ in multiple-band superconductors. In addition, as mention before, an application of Keldysh-Eilenberger model to multiple bands with anisotropic gaps may provide the rigorous description of $\rho_f(B)$ in iron-based materials. We hope that further modifications of our minimal model based on the Keldysh-Eilenberger theory will give more precise picture of $\rho_f(B)$ in multiple-band ($N>3$) superconductors in the clean limit with anisotropic superconducting gaps with inter-band coupling in the future.

C) Two-band model analysis

Fitted results with other gap structures

In the main text, we presented the fitted results with prefactors of $(p_{\mathrm{h}},q_{\mathrm{h}},r_{\mathrm{h}},p_{\mathrm{e}},q_{\mathrm{e}},r_{\mathrm{e}})=(1,0,1,2,1)$, which provide the possibility of horizontal-nodal lines in $\Delta_{\mathrm{h}}(\boldsymbol{k})$ and loop-like nodal lines in $\Delta_{\mathrm{e}}(\boldsymbol{k})$. These prefactors are selected so that the superconducting gaps reflect the symmetry of hole- and electron sheets of Fermi surface. The obtained fitting parameters indicated that the measured data can be reproduced by the combination of a highly anisotropic nodeless gap on the hole-like sheet and a gap with loop-like nodes on the electron-like sheet. To show the validity of these gap structures, we refer to the fitted results with other prefactors leading other gap structures.

Fig.S3**a**-**d** show the calculated results with $(p_{\mathrm{h}},q_{\mathrm{h}},r_{\mathrm{h}},p_{\mathrm{e}},q_{\mathrm{e}},r_{\mathrm{e}})=(1,0,1,1,4,0)$. These prefactors have four-fold in-plane anisotropy and will give eight vertical-nodal lines in $\Delta_{\mathrm{e}}(\boldsymbol{k})$ if $\Delta_{\mathrm{e}}^{\max}\Delta_{\mathrm{e}}^{\min}<0$. The authors of Ref.[19] discussed a similar gap structure and concluded that such vertical nodes are inadequate for explaining the angle-dependence of the thermal conductivity. Meanwhile, Fig.S3**e**-**h** show the results with prefactors of $(1,0,1,1,2,1)$ and with the restrictions of $\Delta_{\mathrm{h}}^{\max}\Delta_{\mathrm{h}}^{\min}<0$ and $\Delta_{\mathrm{e}}^{\max}\Delta_{\mathrm{e}}^{\min}>0$, leading to horizontal-nodal lines in $\Delta_{\mathrm{h}}(\boldsymbol{k})$ and no nodes in $\Delta_{\mathrm{e}}(\boldsymbol{k})$. As shown in Fig.S3, the measured data for $n_s(T)$ and $\rho_f(B)$ were also reproduced by these gap structures with the fit parameters enumerated in Table S1. However, the

residual sum of squares (RSS) of $\Delta_{h,e}$ compared with the ARPES data[20] was the smallest in the case of highly anisotropic nodeless $\Delta_h(\boldsymbol{k})$ and $\Delta_e(\boldsymbol{k})$ with loop-like nodes. Thus, we considered that such a gap structure is realized in $BaFe_2(As_{0.7}P_{0.3})_2$.

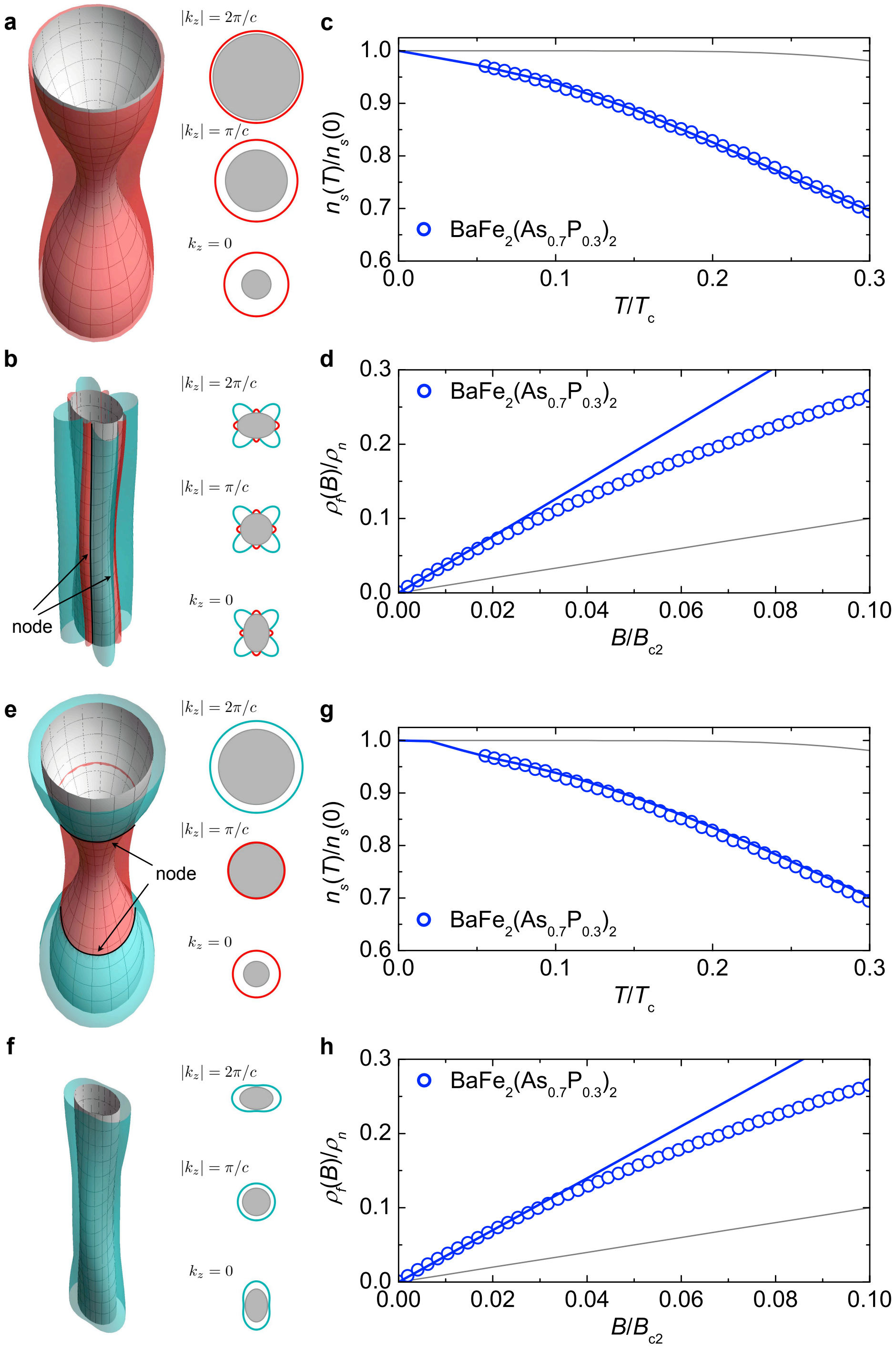

Supplementary Figure S3. **Fitted results of $BaFe_2(As_{0.7}P_{0.3})_2$ for other gap structures.** **a-d**, Results of the fitting with prefactors of $(p_h, q_h, r_h, p_e, q_e, r_e) = (1,0,1,1,4,0)$. **e-h**, Results of the fitting with prefactors of $(1,0,1,1,2,1)$ and with the condition of $\Delta_h^{max}\Delta_h^{min} < 0$, leading to horizontal nodal lines in Δ_h. **a**,**b**,**e**, and **f**, Schematic images of $\Delta(\boldsymbol{k})$ evaluated by the two-band model fitting. **c**,**e**,**g**, and **h**, Calculated results of $n_s(T)/n_s(0)$ (**c**,**e**) and $\rho_f(B)/\rho_n$ (**g**,**h**).

Table S1. Superconducting gaps (in units of $k_B T_c$) estimated by the two-band model analysis. The residual sum of squares of the obtained $\Delta_{h,e}$ compared with those of the ARPES data[20] is also listed.

gap structure	Δ_h^{max}	Δ_h^{min}	Δ_e^{max}	Δ_e^{min}	RSS
ARPES[20]	3.1	1.9	0.8	−3.1	–
nodeless (Δ_h)+loop nodes (Δ_e)	3.1	0.43	1.1	−3.1	2
nodeless (Δ_h)+vertical nodes (Δ_e)	3.7	0.43	0.94	−2.3	3
horizontal nodes (Δ_e)+nodeless (Δ_e)	1.1	−0.79	−0.71	−2.8	14

Resolution of the gap anisotropy

To show the resolution of the two-band model analysis, we present the parameter dependence of the calculated results for LiFeAs and for $BaFe_2(As_{0.7}P_{0.3})_2$ in Figs.S4 and S5, respectively. The black curves, which are the same as the thick curves presented in Fig.1 in the main text, gave the best fit. The red (blue) curves are the results calculated after changing one parameter by +20% (−20%) from the best-fitted value and fixing the other parameters. As an example, we focus on the parameter dependence of the calculated results for LiFeAs (Fig.S4). It is clear that variations of $|\Delta_h^{max}|$ or $|\Delta_h^{min}|$ by ±20% changed $n_s(T)$ drastically, but had nearly no effect on $\rho_f(B)$. In contrast, $\rho_f(B)$ was sensitive to $|\Delta_e^{max}|$ and $|\Delta_e^{min}|$, whereas $n_s(T)$ was insensitive to them. By comparing these results, we can determine $(\Delta_h^{max}, \Delta_h^{min}, \Delta_e^{max}, \Delta_e^{min})$ with errors of less than 10%.

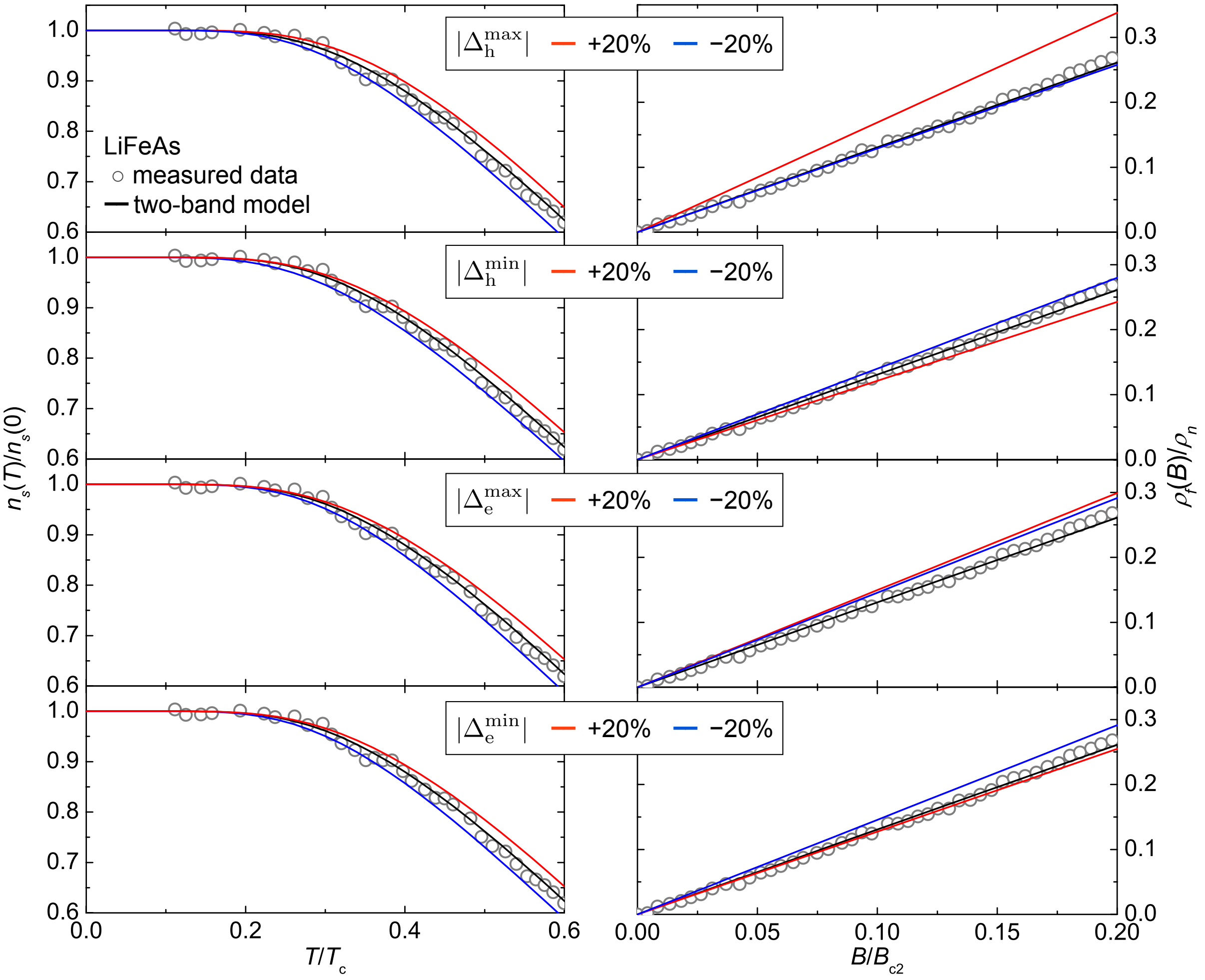

Supplementary Figure S4. Fitting-parameter dependence of LiFeAs. The panels in the left and right columns are the calculated results of $n_s(T)/n_s(0)$ and $\rho_f(B)/\rho_n$, respectively, varying one parameter in each panel: $|\Delta_h^{max}|$, $|\Delta_h^{min}|$, $|\Delta_e^{max}|$, and $|\Delta_e^{min}|$ (top to bottom). The black curves are the best-fitted results, which are the same as the thick curves presented in Fig.1 in the main text. The red (blue) curves are the calculated results with the modification of one parameter by from the best-fitted value +20% (−20%) while fixing the other parameters.

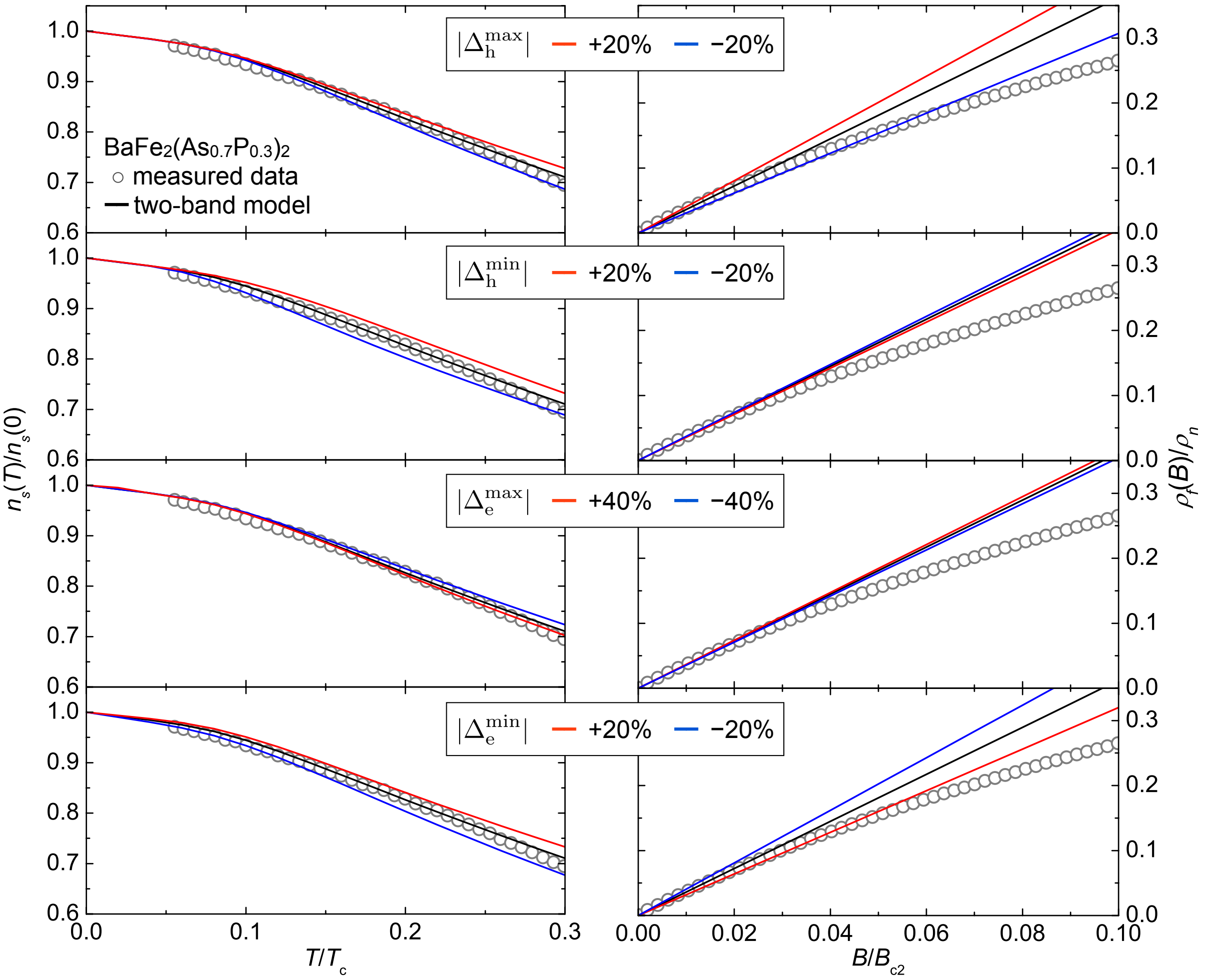

Supplementary Figure S5. **Fitting-parameter dependence of $BaFe_2(As_{0.7}P_{0.3})_2$.** The panels in the left and right columns are the calculated results of $n_s(T)/n_s(0)$ and $\rho_f(B)/\rho_n$, respectively, varying one parameter in each panel: $|\Delta_h^{max}|$, $|\Delta_h^{min}|$, $|\Delta_e^{max}|$, and $|\Delta_e^{min}|$ (top to bottom). The black curves are the best-fitted results, which are the same as the thick curves presented in Fig.1 in the main text. The red (blue) curves are the calculated results with the modification of one parameter by from the best-fitted value +20% (−20%) while fixing the other parameters.

References

1. Okada, T. *et al.* Penetration depth and flux-flow resistivity measurements of $BaFe_2(As_{0.55}P_{0.45})_2$ single crystals. *Phys. C* **504**, 24 (2014).
2. Coffey, M. W. & Clem, J. R. Unified Theory of Effects of Vortex Pinning and Flux Creep upon the rf Surface Impedance of Type-II Superconductors. *Phys. Rev. Lett.* **67**, 386 (1991).
3. Chandrasekhar, B. S. & Einzel, D. The superconducting penetration depth from the semiclassical model. *Ann. Physik* **2**, 535 (1993).
4. Silaev, M. & Vargunin, A. Vortex motion and flux-flow resistivity in dirty multiband superconductors. *Phys. Rev. B* **94**, 224596 (2016).
5. Vargunin, A., Silaev, M. & Babaev, E. Exess magneto-resistance in multiband superconductors due to the viscous flow of composite vortices (2016).
6. Kita, T. Gauge invariance and Hall terms in the quasiclassical equations of superconductivity. *Phys. Rev. B* **64**, 054503 (2001).
7. Goryo, J. & Matsukawa, H. Flux Flow Resistivity in Two-Gap Superconductor. *J. Phys. Soc. Jpn.* **74**, 1394 (2005).

8. Goryo, J., Soma, S. & Matsukawa, H. Vortex state in a two-gap superconductor. *Phys. C* **437**, 86 (2006).

9. Shibata, A. *et al.* Anomalous flux flow resistivity in the two-gap superconductor MgB_2. *Phys. Rev. B* **68**, 060501(R) (2003).

10. Akutagawa, S. *et al.* Quasiparticle Electronic Structure of a New Superconductor, Y_2C_3, in the Mixed State Investigated by Specific Heat and Flux-Flow Resistivity. *J. Phys. Soc. Jpn.* **77**, 064701 (2008).

11. Suhl, H., Matthias, B. T. & Walker, L. R. Bardeen-Cooper-Schrieffer Theory of Superconductivity in The Case of Overlapping Bands. *Phys. Rev. Lett.* **3**, 552 (1959).

12. Zhitomirsky, M. E. & Dao, V. H. Ginzburg-Landau theory of vortices in a multigap superconduct. *Phys. Rev. B* **69**, 054508 (2004).

13. Lin, S. Z. & Bulaevskii, L. N. Dissociation Transition of a Composite Lattice of Magnetic Vortices in the Flux-Flow Regime of Two-Band Superconductors. *Phys. Rev. Lett.* **110**, 087003 (2013).

14. Goryo, J., Soma, S. & Matsukawa, H. Deconfinement of vortices with continuously variable fractions of the unit flux quanta in two-gap superconductors. *Eur. Lett.* **80**, 17002 (2007).

15. Bardeen, J. & Stephen, M. J. Theory of the Motion of Vortices in Superconductors. *Phys. Rev.* **140**, A1169 (1965).

16. Kopnin, N. B. & Volovik, G. E. Flux Flow in *d*-Wave Superconductors: Low Temperature Universality and Scaling. *Phys. Rev. Lett.* **79**, 1377 (1997).

17. Gor'kov, L. P. & Kopnin, N. B. Viscous flow of Vortices in Type-II Superconducting Alloys. *Sov. Phys. JETP* **38**, 195 (1974).

18. Silaev, M. & Babaev, E. Unusual mechanism of vortex viscosity generated by mixed normal modes in superconductors with broken time reversal symmetry. *Phys. Rev. B* **88**, 220504(R) (2013).

19. Yamashita, M. *et al.* Nodal gap structure of superconducting $BaFe_2(As_{1-x}P_x)_2$ from angle-resolved thermal conductivity in a magnetic field. *Phys. Rev. B* **84**, 060507(R) (2011).

20. Yoshida, T. *et al.* Anisotropy of the superconducting gap in the iron-based superconductor $BaFe_2(As_{1-x}P_x)_2$. *Sci. Rep.* **4**, 7292 (2014).
